# Tear proteomics in keratoconus

**Published:** 2010-10-02

**Authors:** Catherine Pannebaker, Heather L. Chandler, Jason J. Nichols

**Affiliations:** College of Optometry, The Ohio State University, Columbus, OH

## Abstract

**Purpose:**

The purpose of this work was to identify potential tear-film based proteins expressed in keratoconus.

**Methods:**

Recruited subjects were normal gas permeable (GP) contact lens wearers, keratoconus subjects wearing GP contact lenses, and keratoconus subjects without contact lenses. Subjects wearing soft lenses or having previous ocular surgeries were excluded from participating. Approximately 5 µl of tears were sampled from both eye of each subject using glass microcapillaries. Additional testing included a brief history, visual acuity, slit lamp examination, and topography. Proteomic analyses used to compare samples included Bradford assays, cytokine arrays, SDS–PAGE, and mass spectrometry.

**Results:**

Forty-four subjects were enrolled in the study including 20 normals (GP wearers), 18 with keratoconus and wearing GPs, and six with keratoconus (non-lens wearers). Across all proteomic approaches, several proteins were identified as possibly being unique to keratoconus. Increased expression of matrix metalloproteinase-1 (MMP-1) was found in keratoconus subjects with and without gas permeable contact lenses (p=0.02). Unique proteins more associated with keratoconus included several keratins, immunoglobulins alpha and kappa, precursors to prolactin, lysozyme C, and lipocalin.

**Conclusions:**

Initial analyses indicate that keratoconus may be associated with the differential expression of several proteins. Further testing is needed to determine any causal relationship or correlation with the etiology of this condition.

## Introduction

Keratoconus is an asymmetric condition of corneal ectasia and thinning with onset usually in early teens to early twenties, with an incidence of about 1/2,000 [1]. The condition can lead to significant visual impairment with high amounts of irregular astigmatism and myopia. Classic objective signs seen by biomicroscopy include corneal stromal thinning, central corneal scarring, vertical lines in the posterior cornea (Vogt’s striae), and prominent corneal nerves; quite often a brownish or olive green colored ring of iron deposition (Fleischer’s ring) is seen at the base of the “cone” or apex of the protrusion [2]. Although improved with pinhole, the best corrected visual acuity in keratoconus subjects is often reduced with spectacle correction; therefore, most subjects are managed with rigid gas permeable (GP) contact lenses in a wide range of specifications. Some subjects may require penetrating keratoplasty if contact lenses are no longer a management option [2].

Keratoconus is historically defined as a non-inflammatory condition [2]. The exact etiology is unknown, however, recent literature suggests that inflammatory molecules and abnormal levels of enzymes are present in subjects with keratoconus [3,4]. Other research indicates that keratoconus may also have genetic components [5]. Frequent associations include history of allergies, atopy (asthma, hay fever, eczema), eye rubbing, eye injuries, rigid or hard contact lens wear, and family history of keratoconus [6]. The condition seems to cease progression with increasing age [7,8].

Extensive tear protein work in subjects without ocular disease performed by de Souza and coworkers [9] has resulted in the identification of 491 proteins, both extracellular and intracellular, the latter of which may result from normal cell death in the epithelium of the cornea. Many proteins are contained in the aqueous layer of the tears and are secreted by the lacrimal and accessory glands in addition to the ocular surface epithilia. The majority of these proteins in the normal tear film consist of lysozyme, lactoferrin, secretory immunoglobulin A, serum albumin, lipocalin, and lipophilin [10]. In addition, these proteins are in a relatively high concentration (8 µg/µl), and easily accessed in tear collection methods, making the tear film very promising for extensive protein analysis.

It is clearly evident that keratoconus is a multifactoral condition. Although it has been historically defined as a noninflammatory condition, recent literature supports a possible role of inflammatory agents in the course of the disease. The aims for this study were to detect tear-film based protein expression differences between keratoconus and normal subjects. This should ultimately start to help further determine the roles of these proteins in the etiology of keratoconus.

## Methods

This study was approved by The Ohio State University Institutional Biomedical Review Board in accordance with the tenets of the Declaration of Helsinki. Written informed consent was obtained by each person before performing the study visit and related procedures.

### Subjects

The subjects recruited were in one of three categories: 1) subjects without a diagnosis of keratoconus wearing GP contact lenses (normals); 2) subjects with a prior diagnosis of keratoconus wearing GP contact lenses; and 3) subjects with a prior diagnosis of keratoconus who did not wear GP or soft contact lenses. Subjects were excluded if they were under 18 years of age, pregnant, currently wearing soft contact lenses, or if there was a history of ocular surgery.

### Clinical exam sequence

The study visit began with a brief history including current age, confirmation of keratoconus condition (or lack thereof), number of years with the diagnosis, and any known family history of keratoconus. Subjects were asked about the length of time wearing GP contact lenses, if applicable, and number of hours the lenses were worn on average per day. Other ocular conditions were noted, along with any systemic conditions and medications being taken.

Best-corrected, high-illumination, high contrast Bailey-Lovie visual acuity was measured independently in each eye, and the number of letters correct was recorded. A tear sample was taken from the inferior tear meniscus of each eye at a biomicroscope while the subject was wearing GP contact lenses (if applicable). A 5-µl glass capillary tube was used (Microcaps; Drummonda Scientfic Co., Broomall, PA.), 2- and 1- μl tubes were used in cases of decreased amount of tears present, which was infrequent. Subjects were instructed to blink normally while obtaining the tear sample, and care was taken by the examiner not to touch the globe to prevent reflex tearing. The volume of the tubes in microliters and the length of the sample in the tube (in millimeters) were recorded so that the approximate volume of the tear samples could be calculated.

Following tear sampling, an assessment of the GP contact lens fit was performed with sodium flourescein and a written filter. Aspects of both the central and peripheral fit were noted, using guidelines associated with the Collaborative Longitudinal Evaluation of Keratoconus (CLEK) Study [6]. The lenses were removed, and slit lamp signs were noted on each subject’s cornea following the removal of contact lenses. The presence or absence of each of the following was noted: Fleisher’s ring, scarring, staining, and Vogt’s Striae. In addition, if any scarring or staining was present on the cornea, grading scales adapted from the CLEK study [6] of density, size and shape were used for quantification in the central, inferior, nasal, temporal, and superior areas. Lastly, topographical maps of each eye were captured using the Orbscan I (Orbtek, Salt Lake City, UT). The steepest and flattest meridians of the simulated keratometry readings were recorded as well as apical radius.

Following the completion of the exam sequence, all tear samples were pushed out from the microcapillaries into microcentrifuge tubes and combined with 20 μl of a reagent consisting of 150 mM NaCl with 50 mM Tris-cl (pH 7.4) and 1 mM EDTA in distilled water. These diluted tear samples were frozen at −80 °C for later analyses.

Before proceeding with testing of the actual samples, a worse-eye classification of the two keratoconus groups was performed based on a variation of the Gold Standard Grading Scheme [11]. This classification method used visual acuity, topography, and presence of slit lamp signs to determine the eye most affected by keratoconus. The worst eye for each keratoconus subject and the right eye of all normal subjects was chosen for tear analyses described below.

### Protein analyses

#### Bradford protein assay

Tear samples were thawed on ice for 15 min and briefly centrifuged to ensure the contents were at the bottom of the tube. A Bradford assay (Quick Start 1×; Bio-Rad Laboratories, Hercules, CA) was run to determine the amount of protein present in individual tear samples. A spectrophotometer (Biomate 3; Thermo Fischer Scientific, Waltham, MA) was use to obtain absorbances as read at 595 nm. Results are reported in μg/μl.

#### RayBio human cytokine antibody array G series

Groups of three samples each were pooled within the three categories of subjects based on similar protein amounts (in μg) for each total volume. The normal subjects consisted of six pooled samples, as did the keratoconus group with gas permeable contact lenses, while the keratoconus group without contact lenses had two pooled samples (due to the limited number of these subjects). The samples were thawed on ice for 15 min and pooled into new microcentrifuge tubes. Twenty microliters from the pooled samples were used for each array. The protocol recommended by the manufacturer was used for the custom RayBio Human Cytokine Antibody Array G Series (Ray-Biotech, Inc., Norcross, GA) with the following modifications: slides were incubated in the blocking buffer for 60 min at room temperature and the Alexa Flour 555-conjugated streptavadin was incubated on the slides overnight at 4 °C before detection.

#### SDS–PAGE gels

Protein samples were mixed with SDS–PAGE loading buffer containing β-mercaptoethanol, heated to 95 °C for 5 min, and subjected to SDS–PAGE analysis. All gels used (18%, 10%, and 6%) were cast in-laboratory. The 18% and 10% gels were run at 175 V for 1.5 h, while the 6% gel was run at 150 V for 1.5 h. After the gels were run, they were removed from the glass plates and fixed in a 40% methanol (MeOH), 10% acetic acid (AcCOOH) solution for 30 min at room termpature on a rocker.

The gels were stained with a 0.2% Coomassie brilliant blue solution (Bio-Rad Laboratories, Hercules, CA) in 45:45:10% MeOH:H2O:AcCOOH for 3 h at room temperature on a rocker. The gels were then destained in a 25:65:10 MeOH:H2O:AcCOOH solution overnight at room temperature to ensure removal of all the background staining, so that only protein bands would appear. Following destaining, the gels were imaged on the Kodak 4000 MM (Carestream Molecular Imaging, Woodbridge, CT), and analyzed using Kodak Molecular Imaging Software (v.4.5.0; Carestream Molecular Imaging) to obtain densitometry readings for all bands.

#### Nano-LC-MS/MS

Capillary-liquid chromatography-nanospray tandem mass spectrometry (nano-LC/MS/MS) was completed for the bands in the 10% gel analysis which displayed evident differences in lane profiling. The 18% and 16% gels exhibited no evidence of difference in bands. A Thermo Finnigan LTQ mass spectrometer (Thermo Fisher Scientific, Inc, Waltham, MA) furnished with a nanospray source was run in positive ion mode. The LC arrangement was an UltiMate™ Plus system from LC-Packings A Dionex Co. (Sunnyvale, CA) equipped with a Famos autosampler and Switchos column switcher (Dionex Co, Sunnyvale, CA). The two solvents used were water with 50 mM acetic acid (Solvent A) and acetonitrile (Solvent B). Each tear sample of five microliters was first instilled on to the trapping column (LC-Packings A Dionex Co, Sunnyvale, CA) and washed with 50 mM acetic acid. The injector port was switched to inject, and the peptides were extracted from the trap onto the column. Chromatographic separations were performed using a 5 cm 75 µm ID ProteoPep II C18 column (New Objective, Inc., Woburn, MA) set directly in the nanospray tip. A gradient of 2%–80%B over 50 min with a flow rate of 300 nl/min was used to extract the peptides from the column into the LTQ system. The total run time was 60 min. The mass spectrometer was programmed for a full scan, using a zoom scan to determine the charge of the peptide, and a MS/MS scan of the most abundant peak in the spectrum. To eliminate multiple MS/MS of the same peptide, dynamic exclusion was used. The MS/MS sequence data was processed using Mascot Distiller to form a peaklist (.mgf file) and by using Mascot MS/MS search engine. Established proteomic guidelines were followed in data processing using a minimum of two sequenced tryptic peptides with a minimum string of five amino acids [12,13]. In addition, assigned peaks had a minimum of 10 counts (signal:noise of three). A 1.5 Da mass accuracy of the precursor ions was set to adjust for unintentional collection of the C13 ion and the fragment mass accuracy was set to 0.5 Da.

### Statistical methods

Variables were first identified as either categorical or continuous and analyzed using frequencies or averages and standard deviations for each of the three groups of subjects, respectively. All continuous variables were then analyzed using the Kruskal–Wallis test for non-parametric data for three-way comparisons, while all two-way comparisons of continuous variables were analyzed using the Mann–Whitney non-parametric test. The Mann–Whitney test was also used as post-hoc testing to confirm significance of findings for the densitometry data from the SDS–PAGE gels.

## Results

### Clinical examination results

The normal group with GP lenses (normals) was 65% female, while the keratoconus group with gas permeable (KCGP) lenses was 16.7% female, and the KC group without GP lenses (KC) had no females. The average age of the normals was 48.3±12.0 years, while it 35.5±13.4 years for the KCGP group, and 37.3±11.5 years for the KC group (p=0.005). The average number of years with a diagnosis of the keratoconus was 8.2±7.5 and 6.7±11.5 years for the KCGP and KC groups, respectively. A family history of keratoconus occurred in 16.7% and 33.3% of the KCGP and KC groups, respectively. The average number of years of wearing time was 26.0±9.8 in the normals and 7.2±7.9 in the KCGP group (p<0.0001). The average number of daily hours of contact lens wearing time in those two groups was 15.2±5.3 (normals) and 12.7±3.3 (KC subjects). With respect to flourescein patterns of contact lenses, nearly 40% of KCGP subjects displayed central touch of some nature, and no normals had central touch. A Fleischer’s ring was present in at least 50% in the two keratoconus groups. The KCGP group had 100% of subjects with striae present, while the KC group had 50% of subjects with striae present. Corneal staining was present in 45% of normal subjects and in over 61% of KCGP group, while none was seen in the KC group. Lastly, the average apical radius found with topography was 43.9±2.7 D in the normals, 52.9±7.3 D in the KCGP group, and 48.6±6.7 D in the KC group (p<0.0001).

### Bradford protein assay results

The tear volume collected at the study visit for normals was 3.13±1.65 µl, 4.30±1.04 µl for the KCGP group, and 4.42±0.69 µl for the KC group (χ^2^=8.20, p=0.02). The total concentration of protein found with the Bradford assay was 7.45±8.28 µg/total volume in the normals, 8.70±4.47 ųg/total volume in the KCGP group, and 9.93±2.80 µg/total volume in the KC group (χ^2^=6.60, p=0.04).

### Cytokine antibody array results

Table 1 displays findings from the cytokine array. As show, matrix metalloproteinase 1 (MMP-1) was not observed in the normal group, but had an average expression level of 34.4±84.4 in the KCGP group and 3,483.4±3,881.0 in the KC group (p=0.02). Although statistically significant results were found for only one of the 40 cytokines tested (at p<0.05), some trends are worth noting. Interleukin-11 (IL-11) had an average expression level of 2,538.9±1,207.1 in the normal group, 1,437.7±840.0 for the KCGP group and 404.1±571.4 for the KC group (p=0.07). Tissue inhibitor of metalloproteinase 1 (TIMP-1) had an average expression level of 7,153.1±2,227.9 in the normal group, 15,579.0±8,213.9 in the KCGP group and 11,898.4±287.5 in the KC group (p=0.06). Tissue inhibitor of metalloproteinase 2 (TIMP-2) also had an average expression level of 22,041.1±5,614.6 in the normal group, 39,824.9±13,027.4 in the KCGP group, and 28,488.2±7,128.8 in the KC group (p=0.10). Lastly, tumor necrosis factor-related apoptosis-inducing ligand receptor 1 (TRAIL R1) had an average expression level of 899.2±992.8 in the normal group, 148.6±301.3 in the KCGP group, and 3,671.7±4,576.3 in the KC group (p=0.09).

**Table 1:** Cytokine array densitometry findings.

**Cytokine**	**Normal group**	**KCGP group**	**KC group**	**χ^2^ test statistic**	**p-value**
EGF	17,306.6±14,098.7	26,900.0±9,397.3	26,926.9±2,395.8	1.68	0.43
EGF-R	586.3±782.2	337.5±666.2	1,957.2±1,825.7	3.11	0.21
FGF-4	0.0±0.0	0.0±0.0	0.0±0.0	N/A	N/A
FGF-6	724.3±1,069.0	1,128.5±1,881.5	688.4±866.8	0.63	0.73
FGF-9	4,074.6±2,401.4	3,879.5±1,405.4	1,542.9±593.9	3.51	0.17
GRO	19,819.8±8,130.2	27,525.7±13,393.4	22,983.2±13,473.3	1.07	0.59
HB-EGF	18.3±44.9	316.3±774.8	1,301.2±1,840.2	1.62	0.44
HGF	1,788.3±1,068.6	1,298.2±744.3	486.4±382.8	2.82	0.24
IL-1 alpha	2,454.5±2,103.1	2,169.2±1,880.8	519.4±734.6	1.23	0.54
IL-1 beta	529.1±545.7	111.6±273.5	382.9±541.6	2.79	0.25
IL-2	3,216.0±2,497.4	1,921.6±1,552.0	4,020.4±5,673.7	1.21	0.55
IL-2 alpha	2,854.8±2,658.6	1,415.3±1,474.0	404.9±572.7	3.38	0.18
IL-2 R beta	2,202.3±1,183.9	1,417.7±873.2	1,039.7±1,023.4	2.21	0.33
IL-2 R gamma	2,090.0±758.3	1,145.7±646.0	1,203.7±1,312.7	4.42	0.11
IL-6	2,683.5±1,527.3	1,228.6±1,337.5	2,508.4±2,004.2	3.28	0.19
IL-6 s R	3,115.7±1,283.0	2,701.8±1,440.6	1,315.2±1,059.2	2.44	0.30
IL-8	3,778.1±1,459.4	8,428.3±14,404.7	1,313.7±1,066.9	3.09	0.21
IL-9	903.9±623.2	2,608.5±4,732.6	774.2±739.0	0.53	0.77
IL-11	2,538.9±1201.7	1,437.7±840.0	404.1±571.4	5.43	0.07
MMP-1	0.0±0.0	34.4±84.4	3,483.4±3,881.0	Z=-2.3*	0.02
MMP-2	1,240.9±1,304.3	778.2±1,309.1	6,325.9±8,019.2	1.99	0.37
MMP-3	1,721.1±946.2	820.3±553.2	1,353.7±1,581.7	2.55	0.28
MMP-9	5,614.4±6504.9	7,513.4±9,602.5	4,415.7±2,622.2	1.37	0.50
MMP-10	629.0±665.1	819.0±1,318.2	2,382.2±2,904.4	1.71	0.43
MMP-13	2,634.6±2,694.4	2,425.1±4,621.1	2,378.4±1,146.8	1.76	0.42
TGF alpha	1,746.7±1,232.0	1,008.2±1,168.0	1,263.9±1,129.2	1.58	0.45
TGF beta	3,164.3±1,402.2	2,118.2±1,275.3	1,506.9±1,645.3	3.35	0.19
TGF beta 2	2,056.1±1,220.5	1,493.1±1,200.3	3,503.4±4,954.6	0.75	0.69
TGF beta 3	1,982.6±1,230.8	1,654.2±1,167.4	2,437.9±2,077.4	0.61	0.74
TIMP-1	7,153.1±2,227.9	15,579.0±8,213.9	11,898.4±287.5	5.64	0.06
TIMP-2	22,041.1±5,614.6	39,824.9±13,027.4	28,488.2±7,128.8	4.61	0.10
TIMP-4	1,470.8±1,043.0	539.2±749.9	1,643.9±1,618.1	2.58	0.28
TNF alpha	3,493.7±3,123.4	1,737.8±1,766.9	1,795.4±531.7	1.09	0.58
TNF beta	4,650.1±3,291.8	2,263.2±1,418.8	1,140.7±1,276.6	2.42	0.30
TNFRSF6/Fas	386.7±401.4	97.6±109.7	1,139.3±1,611.2	1.42	0.49
TRAIL R1	899.2±992.8	148.6±301.3	3,671.7±4,576.3	4.82	0.09
uPAR	3,057.3±1,648.1	2,219.0±1,062.6	1,184.4±906.8	3.07	0.22
VEGF	1,957.7±1,129.1	1,054.3±1,157.0	1,063.4±1,503.9	1.77	0.41
VEGF-D	1,257.4±2,014.7	176.2±302.2	1,442.2±1,782.5	3.51	0.17
VEGF R2	3,062.1±1,109.8	2,160.0±1,366.4	2,981.9 ±2,889.9	1.11	0.58

### SDS–PAGE gel results

**Figure 1:**
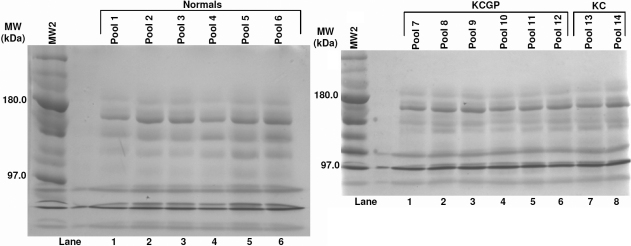
6% 1D-SDS–PAGE results.

**Figure 2:**
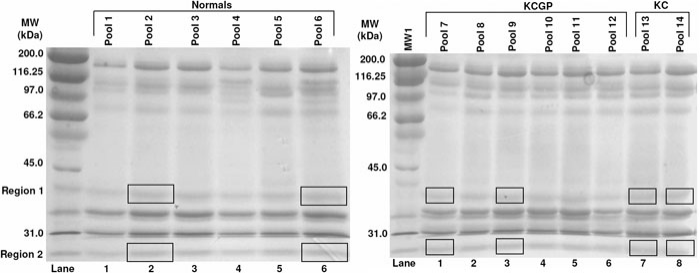
10% 1D-SDS–PAGE results.

**Figure 3:**
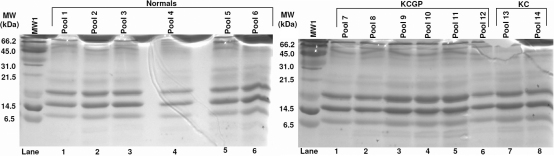
18% 1D-SDS–PAGE results.

No bands of interest or difference were found in the 6% gel (Figure 1, Table 2). As summarized in Figure 2 and Table 3, band 4 of the 10% gel showed significant results with post hoc testing; density averages of 4,267,008±926,704 were seen in the normal group, 2,712,487±1,214,430 was seen in the KC group with lenses, and 3,143,803±1,068,539 in the KC group without lenses (Kruskal–Wallis [KW] p=0.06, Mann–Whitney [MW] p=0.03). Because bands 7, 9, and 10 in the 6% gel had unreliable data, no results are shown for these bands. The 10% gel revealed statistically significant densitometries in three different bands. The reading in band 6 for the normals was 2,432,324±520,023, 3,195,882±631,948 in the KC group with lenses and 3,731,696±2,191,853 in the KC group without lenses. Results in band 8 in the normals showed an average of 2,378,994±476,567 in the normals, 4,338,343±942,065 in the KC group with lenses, and 4616,313±2,598,085 in the KC group without lenses (KW p=0.017, MW p=0.010). Band 10 density averages were 2,981,027±669,225 in normals, 4,172,691±672,334 in the KC group with lenses, and 3,895,599±2,279,192 in the KC group without lenses (KW p=0.074, MW p=0.037). Bands 8 and 10 in the 10% gel were further analyzed with mass spectrometry given the level of significance in difference in band intensities across the three groups. In the 18% gels (Figure 3 and Table 4), band 2 had significant readings after post-hoc testing. The normals had an average reading of 5,092,928±1,766,856, while the KC group with lenses read 2,465,169±1,653,506, and the KC group without lenses read 1,653,506±139,621 (KW p=0.074, MW p=0.037).

**Table 2:** Results of 6% SDS–PAGE gel.

Band*	**Normal group**	**KCGP group**	**KC group**	**χ^2^ test statistic**	K-W** p-value	M-W*** p-value
1	3,575,887±1,424,689	1,806,129±217779	2,591,190±2,831,972	3.35	0.19	0.06
2	2,077,964±286,289	2,181,524±234,306	2,913,398±1,655,816	0.42	0.81	0.52
3	5,872,456±1,330,132	4,859,815±572,571	6,273,139±1,202,384	4.15	0.13	0.11
4	4,267,008±926,704	2,712,487±1,214,430	3,143,803±1,068,539	5.49	0.06	0.04
5	2,649,799±769,856	2,553,806±360,750	3,312,287±1,033,510	1.20	0.55	1.00
6	1,753,659±502,052	1,919,784±520,070	2,462,628±496,940	1.87	0.39	0.63
8	3,032,929±346,532	3,227,170±169,248	3,986,479±736,975	5.73	0.06	0.26

Bands 7, 9, and 10 had unreliable data. **Kruskal–Wallis non-parametric three-way analysis testing performed. ***Mann–Whitney non-parametric two-way analysis performed on normals w/ GPs and KC w/ GPs (pools 1–12).

**Table 3:** Results of 10% SDS–PAGE gel.

**Band**	**Normal group**	**KCGP group**	**KC group**	**χ^2^ test statistic**	K-W* p-value	M-W* p-value
1	6,670,481±1,317,847	7,576,569±1,778,475	9,230,756±5,269,558	3.77	0.15	0.34
2	3,168,342±531,197	2,748,752±882,552	3,306,052±1,713,673	1.91	0.39	0.26
3	4979785±1,106,348	4,350,204±979,721	4,951,500±2,808,472	1.47	0.48	0.34
4	3,326,409±617,688	3,362,000±1,716,671	5,329,212±2,554,452	2.15	0.34	1.00
5	2,954,540±691,520	3,462,319±813,563	5,226,575±3,120,471	5.73	0.06	0.26
6	2,432,324±520,023	3,195,882±631,948	3,731,696±2,191,853	6.55	0.04	0.06
7	6,879,351±1,396,260	6,939,410±800,908	7,019,099±4,396,925	0.34	0.84	0.87
8	2,378,994±476,567	4,338,343±942,065	4,616,313±2,598,085	8.15	0.02	0.01
9	8,374,828±1,871,081	9,312,139±303,743	9,651,734±6,610,028	1.20	0.55	0.87
10	2,981,027±669,225	4,172,691±672,334	3,895,599±2,279,192	6.71	0.04	0.03

**Kruskal–Wallis non-parametric three-way analysis testing performed. ***Mann–Whitney non-parametric two-way analysis performed on normals w/ GPs and KC w/ GPs (pools 1–12).

**Table 4:** Results of 18% SDS–PAGE gel.

**Band**	**Normal group**	**KCGP group**	**KC group**	**χ^2^ test statistic**	K-W* p-value	M-W* p-value
1	4,735,056±3,714,884	2,936,292±2,062,411	2,062,411±731,122	2.46	0.29	0.26
2	5,092,928±1,766,856	2,465,169±1,653,506	1,653,506±139,621	5.22	0.07	0.04
3	5,175,055±1,448,604	7,423,089±1,473,792	1,473,792±478,052	5.87	0.05	0.06
4	6,929,268±2,110,943	8,659,253±1,024,358	1,024,358 ±1,200,761	5.41	0.07	0.20
5	3,738,795±2,209,871	5,101,108±1,482,155	1,482,155±261,301	5.73	0.06	0.26

**Kruskal–Wallis non-parametric three-way analysis testing performed. ***Mann–Whitney non-parametric two-way analysis performed on normals w/ GPs and KC w/ GPs (pools 1–12).

### Mass spectrometry results

The proteins were identified using nano-liquid chromatography-tandem mass spectrometry (nano-LC-MS/MS) from two regions of the 10% gel of the SDS–PAGE gel. Proteins found to be more associated with the normal group were immunoglubulin (Ig) alpha-1, Ig lambda, and Ig kappa. Proteins identified in both groups of keratoconus subjects from pools were keratin types I and II, neutrophil-defensin 1 precursor, and mammaglobulin-B precursor. Numerous proteins common to all groups of subjects included lactotransferrin, lysozyme C presursor, lacritin, lipocelin, prolactin-inducible protein, and proline-rich 4.

## Discussion

Although keratoconus is classically defined as a noninflammatory condition, there appears to be growing evidence suggesting that inflammation is associated with the condition. For example, elevated serum levels of IgE, IgG, and IgM have been reported in keratoconus [14,15]. More recently, Lema and Durán [4] targeted specific cytokines, cell adhesion molecules, and proteases in patients with keratoconus, and found levels of IL-6, TNF-α, and MMP-9 higher in keratoconus subjects as compared to normals. Li and Pflugfelder [16] reported that MMP-9 may be involved in corneal inflammation. Lema made a compelling remark: “It can be concluded…that keratoconus cannot be defined any more as a noninflammatory disorder” [4].

Collier [17] addressed MMPs and their possible role in keratoconus. He specifically addressed the absence of upregulation of MMP-9 in prior studies by suggesting that techniques could be a possible source of conflict [17-20]. Collier additionally notes that MMP-9 can be induced by IL-1, and others have noted that keratoconus fibroblasts release fourfold the number of these same IL-1 receptors compared to normal corneas [17,21]. It would stand to reason that MMP-9 could indeed be overexpressed in keratoconus.

It is important to note that the cornea is 70% collagen by weight [17]. The ectasia and thinning found in keratoconus is mostly due to a damaged extracellular matrix and a decrease in types I and IV collagen [17,22]. Levels of telopeptides, or collagen degradation products, were studied by Abalian [22] and found to be 3.5 times higher in keratoconus patients compared to normals; contact lens wear did not significantly modify the amounts.

Kenney et al. [3] described a “cascade hypothesis of keratoconus” in which enzymes could possibly lead to oxidative damage by altering corneal proteins and ultimately lead to apoptosis, altered signaling pathways, increased enzyme activities and fibrosis. These cytotoxic agents in keratoconus corneas may lead to corneal thinning and loss of vision [3,23]. This hypothesis is supported by evidence showing that the inhibitors of destructive enzymes are decreased in keratoconus corneas; they are alpha one (α1 proteinase inhibitor, alpha two (α2) macroglobulin, and tissue inhibitor metalloproteinase one (TIMP-1); the latter of which can inhibit cell apoptosis [3,20,24,25]. Kenney et al. [3,23] surmised that reactive oxygen species may lead to large amounts of cytotoxic by products in keratoconus corneas eventually leading to corneal thinning and loss of vision.

Corneal scarring is significant in keratoconus because it leads to a reduction in transparency and visual impairment [26]. In a normal cornea, the epithelium is constantly renewed and the stroma is in a state of deturgescence; however, layers such as the endothelium or basement membrane are compromised, a repair response is initiated by various growth factors and cytokines which contribute to fibrotic tissue [26,27]. Transforming growth factor beta (TGFβ) is important in ocular scar development in activating macrophages, corneal fibroblasts, and other fibrosis-related growth factors [26]. This repair response causes the cornea to lose transparency due to the disorganization of the fibrotic repair tissue [27].

### Cytokine antibody array

The results of the cytokine antibody arrays revealed a statistically significant increase in matrix metalloproteinase-one (MMP-1) in the tears of both KC groups. No MMP-1 was found in the normal group. MMP-1 is a member of a family of enzymes which break down components of the extracellular matrix, and its specific substrates are corneal collagens type I and III [16.28]. MMP-1 should have minimal to no expression in healthy tissue and is involved in vascularization, wound healing, and inflammatory processes [16,28]. MMP-1 was found to be increased in the corneas of keratoconus subjects by Seppälä et al. [29]. Our study may be one of the first to show the presence of MMP-1 in the tears of keratoconus subjects.

Another trend found was an increase in tissue inhibitor of metalloproteinase 1 (TIMP-1) in the keratoconus subjects compared to normals. TIMP-1 exhibits anti-apoptotic properties and inhibits several MMPs [30,31]. Kenney and coworkers [31] suggested that lower levels of TIMP-1 may lead to the corneal degradation found in keratoconus corneas, and found that 32 keratoconus corneas exhibited a 1.8 fold decrease in TIMP-1 as detected by western-blot analysis. Matthews and coworkers [30] found that TIMP-1 protected against corneal apoptosis in cultured corneal stromal cells injected with adenoviral vectors and quantified by ELISA. Another study to note by Smith and coworkers [32] showed a lack of increase of TIMP-1 in clear keratoconus corneas and yet an significant increase of TIMP-1 in scarred keratoconus corneas, and suggested that TIMP-1 may play a role in “curtailing keratoconus.” The results of our study seem to indicate that further testing is needed to verify the presence and function of TIMP-1 in tears in keratoconus patients.

A final trend to be noted in our study from the arrays was with tumor necrosis-related apoptosis-inducing ligand-R1 (TRAIL-R1).Tumor cell apoptosis is initiated when this receptor binds the TRAIL ligand, however, it spares normal cells [33-36](36). In our study, TRAIL-R1 was reduced in the KC group with GP lenses and increased in the KC group without lenses as compared to normals. This suggests the possibility that in keratoconus these receptors are inappropriately expressed which results in cell death. Because we measured TRAIL-R1 in tears, several ocular surface structures could be considered the source of the ligand. There appears to be no published literature to date linking TRAIL-R1 to keratoconus, or any related functions in the cornea or tears. Further testing could explore the potential upregulation of TRAIL-R1 in the early phases of keratoconus. Although the trends noted are intriguing, conclusions are tentative given the sample size and gender distribution.

### SDS–PAGE gels and mass spectrometry

The bands of interest in the 10% SDS–PAGE gels produced curious results when identified by nano-LC-MS/MS in that cytoskeletal keratins were found to be present in the tear samples in both groups of keratoconus subjects. Keratins are normally found in the outermost layer of the epidermis and not necessarily in tears (unless contaminated from the eyelids due to eye rubbing), while the epithelium of the cornea is non-keratinized. Nakamura and coworkers reported increased cytokeratins in ocular surface-diseased corneas and found keratins in diseased conjunctival cells of Sjogrens and ocular cicatrical pemphigoid subjects by immunohistochemical studies [37,38]. In related research, Dogru and coworkers [39] found a relationship between severity of keratoconus and the degree of conjunctival squamous metaplasia. These reports plus our findings suggest several ocular surface disorders are associated with pathologic keratinization. A protein that was only found by mass spectrometry only in the keratoconus subjects was a precursor to mammaglobin B. Mammaglobin B is a gene expressed in tumors of the esophagus, stomach, colon, pancreas, common bile duct, cholangioma and gall bladder, and is increased in breast cancer [40,41]. Molloy and coworkers [41] identified a protein in tears called lacryglobin, whose sequence of amino acids is identical to 68 of those seen in mammaglobin. Lacryglobin has been seen in the tears of some cancer subjects [42]. Mammaglobin B is also known as secretoglobin 2A1 which has expression in many ocular glands. It has been suggested that it binds hydrophobic ligands and may play a role in tear film lipid layer formation, as well as androgen-deficient disorders such as severe dry eye in Sjogrens Syndrome [43].

Initial analyses in this study shows that tear proteomic techniques can assist in etiologic studies of keratoconus. The differential expression of tear film proteins such as MMP-1, keratins, and mammaglobin B can be found in keratoconus subjects. These findings suggest that further testing could help determine if these molecules have a role in the etiology of keratoconus.
